# A Machine Learning Approach for the Prediction of Traumatic Brain Injury Induced Coagulopathy

**DOI:** 10.3389/fmed.2021.792689

**Published:** 2021-12-10

**Authors:** Fan Yang, Chi Peng, Liwei Peng, Jian Wang, Yuejun Li, Weixin Li

**Affiliations:** ^1^Department of Plastic Surgery and Burns, Tangdu Hospital, Fourth Military Medical University, Xi'an, China; ^2^Department of Health Statistics, Second Military Medical University, Shanghai, China; ^3^Department of Neurosurgery, Tangdu Hospital, Fourth Military Medical University, Xi'an, China

**Keywords:** traumatic brain injury-induced coagulopathy, TBI-IC, machine learning, external validation, model interpretation

## Abstract

**Background:** Traumatic brain injury-induced coagulopathy (TBI-IC), is a disease with poor prognosis and increased mortality rate.

**Objectives:** Our study aimed to identify predictors as well as develop machine learning (ML) models to predict the risk of coagulopathy in this population.

**Methods:** ML models were developed and validated based on two public databases named Medical Information Mart for Intensive Care (MIMIC)-IV and the eICU Collaborative Research Database (eICU-CRD). Candidate predictors, including demographics, family history, comorbidities, vital signs, laboratory findings, injury type, therapy strategy and scoring system were included. Models were compared on area under the curve (AUC), accuracy, sensitivity, specificity, positive and negative predictive values, and decision curve analysis (DCA) curve.

**Results:** Of 999 patients in MIMIC-IV included in the final cohort, a total of 493 (49.35%) patients developed coagulopathy following TBI. Recursive feature elimination (RFE) selected 15 variables, including international normalized ratio (INR), prothrombin time (PT), sepsis related organ failure assessment (SOFA), activated partial thromboplastin time (APTT), platelet (PLT), hematocrit (HCT), red blood cell (RBC), hemoglobin (HGB), blood urea nitrogen (BUN), red blood cell volume distribution width (RDW), creatinine (CRE), congestive heart failure, myocardial infarction, sodium, and blood transfusion. The external validation in eICU-CRD demonstrated that adapting boosting (Ada) model had the highest AUC of 0.924 (95% CI: 0.902–0.943). Furthermore, in the DCA curve, the Ada model and the extreme Gradient Boosting (XGB) model had relatively higher net benefits (ie, the correct classification of coagulopathy considering a trade-off between false- negatives and false-positives)—over other models across a range of threshold probability values.

**Conclusions:** The ML models, as indicated by our study, can be used to predict the incidence of TBI-IC in the intensive care unit (ICU).

## Introduction

Traumatic brain injury (TBI) is still one of the leading causes of death and disability worldwide with over 10 million people hospitalized every year (1). It is common to witness the alterations of the coagulative system and disturbed coagulation function in TBI patients. Results from previous studies indicated that two in three patients with severe TBI manifested coagulation system abnormalities upon admission to the emergency department, and then continued to worsen (2, 3). And the overall mortality of TBI-induced coagulopathy (TBI-IC) attains 17–86% (4–6). TBI-IC is characterized by both hypo-coagulopathy with prolonged bleeding or hyper-coagulopathy with an increased prothrombotic tendency, or both (4, 7). Previous study unearthed that coagulopathy following TBI was related to higher mortality and prolonged intensive care unit (ICU) stay (8). In early stage, potential mechanisms include the dysfunction of the coagulation cascade and hyperfibrinolysis, both of which contribute to hemorrhagic progression. Later, a poorly defined prothrombotic stage emerges, partly caused by fibrinolysis shutdown and hyperactive platelets (9–11).

Undoubtedly, it is imperative to promote the early identification of TBI-IC in a timely way. Laboratory assays, including international normalized ratio (INR) and thromboelastogram are widely used to diagnose TBI-IC. Nonetheless, these assays have limited value in predicting coagulopathy before it develops. In recent years, as a field of artificial intelligence, machine learning (ML) is able to learn from data based on computational modeling. Likewise, ML can fit high-order relationships between covariates and outcomes in data-rich environments (12–14).

This study aimed to determine whether ML algorithms using demographic, comorbidities, laboratory examinations and other variables could predict TBI-IC with considerable accuracy and identify factors contributing to the prediction power.

## Materials and Methods

### Data Source

We conducted this retrospective study based on two sizeable critical care databases, the Medical Information Mart for Intensive Care (MIMIC)-IV version 1.0 (15) and eICU Collaborative Research Database (eICU-CRD) version 1.2 (16). In brief, the MIMIC-IV database, an updated version of MIMIC-III, incorporated comprehensive, de-identified data of patients admitted to the ICUs at the Beth Israel Deaconess Medical Center in Boston, Massachusetts, between 2008 and 2019, containing data from 383220 distinct admissions (single center). The other database, eICU-CRD, was a multicenter, freely available, sizeable database with de-identified high granularity health data associated for over 200,000 admissions to ICUs across the United States between 2014 and 2015. This study was approved by the Institutional Review Boards of Beth Israel Deaconess Medical Center (Boston, MA) and the Massachusetts Institute of Technology (Cambridge, MA). Requirement for individual patient consent was waived because the study did not impact clinical care and all protected health information was deidentified. One author (CP) has obtained access to both databases and was responsible for data extraction (Certification number: 41657645). The study was reported in accordance to the REporting of studies Conducted using Observational Routinely collected health Data (RECORD) statement (17).

### Participant Selection

Inclusion criteria were patients with moderate and severe TBI [msTBI: defined as Glasgow Coma Score (GCS) = < 12]. People with an age of less than 16 years old, ICU stays less than 48 h, and no coagulation index within 24 h of ICU admission were excluded from the study. Moreover, for patients with ICU admissions more than once, only data of the first ICU admission of the first hospitalization were included in the analysis.

### Predictors of Coagulopathy

A total of 53 predictor variables for the ML models were initially included. Specifically, in this study, the data were extracted from MIMIC-IV and eICU-CRD including age, gender, race, family history of stroke. Coexisting disorders were also collected based on the recorded International Classification of Diseases (ICD)-9 and ICD-10 codes. Then, the Charlson comorbidity index (CCI) was calculated from its component variables [myocardial infarction, congestive heart failure, peripheral vascular disease, cerebrovascular disease, dementia, chronic pulmonary disease, rheumatic disease, peptic ulcer disease, diabetes, paraplegia, renal disease, malignant cancer, severe liver disease, metastatic solid tumor and acquired immunodeficiency syndrome (AIDS)]. Lastly, we extracted data containing vital signs, laboratory findings, injury type, different therapy strategies and scoring system on the first day of ICU admission. Details of missing data can be seen in Supplementary Table 1.

### Outcome

In accordance to previous literature, the following parameters were considered for diagnosing coagulopathy: an activated partial thromboplastin time (APTT) > 40s, an INR > 1.4, or platelet (PLT) counts < 100 × 10^9^/L (4, 18).

### Statistical Analysis

Values were presented as the means with standard deviations (if normal) or medians with interquartile ranges (IQR) (if non-normal) for continuous variables, and total numbers with percentages for categorical variables. Proportions were compared using χ^2^ test or Fisher exact tests while continuous variables were compared using the *t* test or Wilcoxon rank sum test, as appropriate.

**Figure 1:**
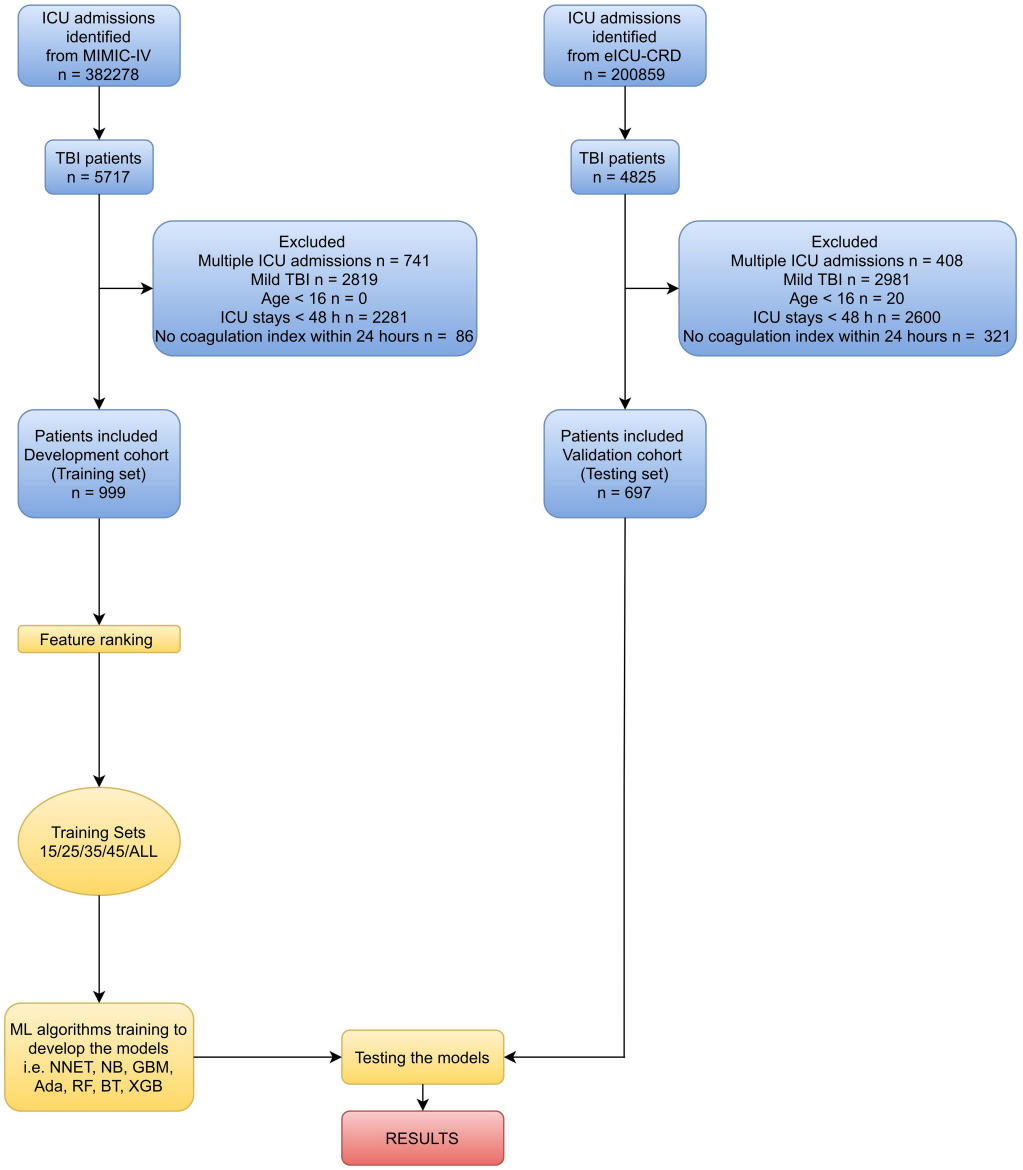
Overview of the methods used for data extraction, training, and testing. ICU, intensive care unit; MIMIC-IV, Medical Information Mart for Intensive Care-IV; eICU-CRD, eICU Collaborative Research Database; TBI, traumatic brain injury; ML, machine learning; NNET, artificial neural network; NB, naïve bayes; GBM, gradient boosting machine; Ada, adapting boosting; RF, random forest; BT, bagged trees; XGB, eXtreme Gradient Boosting.

In this study, recursive feature elimination (RFE) as a feature selection method was used to select the most relevant features. In short, RFE recursively fits a model based on smaller feature sets until a specified termination criterion is reached. In each loop, in the trained model, features were ranked based on their importance. Finally, dependency and collinearity were eliminated. Features were then considered in groups of 15/25/35/45/ALL (ALL = 53 variables, as represented in Figure 1) organized by the ranks obtained after the feature selection method. To find the optimal hyperparameters, 10-fold cross-validation was used as a resampling method. In each iteration, every nine folds were used as training subset, and the remaining one fold was processed to tune the hyperparameters. This training-testing process was repeated thirty times. And in this way, each sample would be involved in the training model, and also participated in the testing model, so that all data were used to the greatest extent.

In this study, we employed seven diverse ML algorithms to develop models, containing artificial neural network (NNET), naïve bayes (NB), gradient boosting machine (GBM), adapting boosting (Ada), random forest (RF), bagged trees (BT), and eXtreme Gradient Boosting (XGB). Initially, we conducted internal validation on the development sets to quantify optimism in the predictive performance and evaluate stability of the prediction model. Bootstrap Resampling technique with 100 iterations was used to evaluate the internal validity of each model. External validation of the models was performed in eICU-CRD. All the models were assessed in multiple dimensions regarding their model performance. The median and 95% confidence intervals of area under the curve (AUC) were calculated, where an AUC value of 1.0 means perfect discrimination and 0.5 represents no discrimination. And the accuracy, sensitivity, specificity, negative predictive value, and positive predictive value were also calculated. Additionally, to determine the clinical usefulness of the included variables by quantifying the net benefit at different threshold probabilities, we conducted the decision curve analysis (DCA) (19). Finally, the “Shiny” package in the R was used to construct a visual data analysis platform.

All analyses were performed by the statistical software packages R version 4.0.2 (http://www.R-project.org, The R Foundation). In our study, we used the “Caret” R packages to achieve the process. *P* values less than 0.05 (two-sided test) were considered as statistically significant.

## Results

### Baseline Characteristics

Variable values on the first day of the TBI patients in MIMIC-IV were analyzed. As shown in Figure 1 and Table 1 of 5717 TBI patients in MIMIC-IV, 999 were included in the final cohort. A total of 493 patients developed coagulopathy, whereas 506 patients did not. A cohort of 285 patients with coagulopathy following TBI in eICU-CRD was included as external dataset (Supplementary Table 2). The process of data extraction, training preparation, data testing via different ML algorithms is depicted in Figure 1. People who had coagulopathy were more likely to be female, with family history of stroke, myocardial infarction, congestive heart failure, peripheral vascular disease, cerebrovascular disease, renal disease, malignant cancer, severe liver disease, metastatic solid tumor as well as having higher CCI, heart rate, respiratory rate, red blood cell volume distribution width (RDW), INR, lactate, buffer excess (BE), FiO_2_, chloride, sodium, glucose, creatinine (CRE), blood urea nitrogen (BUN), blood transfusion, sepsis related organ failure assessment (SOFA), acute physiology score III (APSIII), and longer APTT, prothrombin time (PT), mechanical ventilation (MV). Furthermore, they were less likely to have dementia, cerebral contusion, with lower temperature, mean artery pressure (MAP), red blood cell (RBC), white blood cell (WBC), hemoglobin (HGB), PLT, hematocrit (HCT), pH, bicarbonate, PaO_2_/FiO_2_, calcium, urine output, and GCS.

**Table 1:** Baseline characteristics of the MIMIC-IV cohorts.

	**MIMIC-IV**		
**Variables**	**Coagulopathy (*n* = 493)**	**Non-Coagulopathy (*n* = 506)**	***P*** **Value**
Demographics			
Age (y), median [Q1, Q3]	67.00 (52.00, 80.00)	66.00 (48.00, 82.00)	0.809
Male, *n* (%)	317 (62.65)	343 (69.57)	0.025
Race, *n* (%)			
Black	24 (4.87)	28 (5.53)	
White	294 (59.63)	304 (60.08)	
Hispanic	14 (2.84)	16 (3.16)	
Asian	15 (3.04)	10 (1.98)	
Others	146 (29.61)	148 (29.25)	
BMI (kg/m^2^), median [Q1, Q3]	26.25 (23.03, 29.80)	26.12 (23.10, 30.10)	0.914
Family history of stroke, *n* (%)	19 (3.85)	5 (0.99)	0.006
Coexisting disorders, *n* (%)			
Myocardial infarction	69 (14.00)	23 (4.55)	<0.001
Congestive heart failure	122 (24.75)	44 (8.70)	<0.001
Peripheral vascular disease	39 (7.91)	20 (3.95)	0.012
Cerebrovascular disease	98 (19.88)	75 (14.82)	0.043
Dementia	22 (4.46)	45 (8.89)	0.008
Chronic pulmonary disease	75 (15.21)	61 (12.06)	0.173
Rheumatic disease	11 (2.23)	5 (0.99)	0.189
Peptic ulcer disease	12 (2.43)	5 (0.99)	0.128
Diabetes	108 (21.91)	122 (24.11)	0.452
Paraplegia	47 (9.53)	62 (12.25)	0.202
Renal disease	73 (14.81)	42 (8.30)	0.002
Malignant cancer	36 (7.30)	10 (1.98)	<0.001
Severe liver disease	23 (4.67)	0 (0.00)	<0.001
Metastatic solid tumor	10 (2.03)	2 (0.40)	0.038
AIDS	2 (0.41)	2 (0.40)	1.000
CCI, median [Q1, Q3]	5.00 (3.00, 7.00)	4.00 (2.00, 6.00)	<0.001
Vital signs (1st 24h)			
Temperature (°C), median [Q1, Q3]	37.10 (36.70, 37.50)	37.20 (36.90, 37.52)	0.017
MAP (mmHg), median [Q1, Q3]	79.00 (73.00, 86.00)	81.00 (76.00, 88.00)	<0.001
Heart rate (min), median [Q1, Q3]	86.00 (76.00, 99.00)	84.00 (73.00, 95.00)	0.004
Respiratory rate (min), median [Q1, Q3]	19.00 (17.00, 22.00)	18.00 (16.00, 20.00)	<0.001
Laboratory findings (1st 24h)			
RBC (10^9^/L), median [Q1, Q3]	3.40 (3.00, 3.80)	3.80 (3.30, 4.20)	<0.001
WBC (× 10^9^/L), median [Q1, Q3]	11.60 (8.33, 14.80)	12.30 (9.50, 15.00)	0.029
HGB (g/dL), median [Q1, Q3]	11.00 (9.00, 12.00)	12.00 (10.00, 13.00)	<0.001
PLT (× 10^9^/L), median [Q1, Q3]	166.50 (119.00, 224.75)	219.00 (178.00, 265.00)	<0.001
RDW (%), median [Q1, Q3]	17.20 (14.50, 47.80)	15.50 (13.60, 44.98)	<0.001
HCT (%), median [Q1, Q3]	31.90 (27.83, 35.77)	35.10 (31.20, 38.40)	<0.001
APTT (s), median [Q1, Q3]	31.40 (27.70, 38.20)	27.60 (25.70, 30.17)	<0.001
PT (s), median [Q1, Q3]	15.40 (13.40, 17.90)	12.90 (12.00, 13.80)	<0.001
INR, median [Q1, Q3]	1.40 (1.20, 1.60)	1.20 (1.10, 1.20)	<0.001
pH, median [Q1, Q3]	7.39 (7.34, 7.43)	7.40 (7.37, 7.44)	0.001
Bicarbonate (mmol/L), median [Q1, Q3]	22.50 (20.00, 25.00)	23.30 (21.00, 25.00)	0.002
Lactate (mmol/L), median [Q1, Q3]	1.80 (1.20, 2.60)	1.50 (1.00, 2.12)	<0.001
BE (mEq/L), median [Q1, Q3]	−0.71 (-3.00, 1.00)	0.00 (-1.50, 1.50)	<0.001
Anion gap (mmol/L), median [Q1, Q3]	14.80 (12.80, 16.70)	14.50 (13.00, 16.30)	0.467
PaO_2_ (mmHg), median [Q1, Q3]	141.48 (104.91, 191.65)	148.33 (103.25, 193.69)	0.560
PaCO_2_ (mmHg), median [Q1, Q3]	38.33 (35.00, 42.67)	38.46 (35.00, 43.00)	0.784
FiO_2_ (%), median [Q1, Q3]	50.00 (42.50, 60.00)	50.00 (40.00, 57.50)	0.025
PaO_2_/FiO_2_, median [Q1, Q3]	286.29 (208.26, 372.00)	313.72 (227.25, 413.23)	0.008
Chloride (mmol/L), median [Q1, Q3]	105.50 (102.00, 109.30)	104.50 (101.00, 108.00)	0.001
Calcium (mmol/L), median [Q1, Q3]	8.30 (7.80, 8.70)	8.50 (8.00, 8.90)	<0.001
Sodium, (mmol/L), median [Q1, Q3]	140.00 (137.00, 142.80)	140.00 (137.00, 141.80)	0.049
Potassium (mmol/L), median [Q1, Q3]	4.10 (3.80, 4.40)	4.00 (3.80, 4.30)	0.197
Glucose (mmol/L), median [Q1, Q3]	141.00 (116.00, 166.00)	133.00 (114.50, 159.00)	0.035
CRE (mg/dL), median [Q1, Q3]	1.00 (0.70, 1.30)	0.90 (0.70, 1.10)	<0.001
BUN (mg/dL), median [Q1, Q3]	17.50 (12.30, 26.70)	15.00 (11.00, 20.00)	<0.001
Urine output (mL), median [Q1, Q3]	1668.00 (1078.00, 2462.50)	1875.00 (1250.00, 2673.75)	0.018
Type of injury, *n* (%)			
Subarachnoid hemorrhage	175 (35.50)	162 (32.02)	0.273
Cranial extradural hematoma	18 (3.65)	16 (3.16)	0.801
Cerebral contusion	74 (15.01)	124 (24.51)	<0.001
Therapy strategy (1st 24h), *n* (%)		
MV	436 (88.44)	401 (79.25)	<0.001
Blood Transfusion	29 (5.88)	5 (0.99)	<0.001
Hyperosmolar therapy	46 (9.33)	63 (12.45)	0.139
Neurosurgical intervention	146 (29.61)	153 (30.24)	0.884
Scoring system			
GCS	8.00 (5.00, 10.00)	8.00 (7.00, 10.00)	0.001
SOFA	7.00 (5.00, 10.00)	5.00 (4.00, 6.00)	<0.001
APSIII	39.00 (31.00, 48.00)	35.00 (27.00, 43.00)	<0.001

*MIMIC-IV, Medical Information Mart for Intensive Care-IV; BMI, body mass index; AIDS, acquired immunodeficiency syndrome; CCI, Charlson comorbidity index; MAP, mean artery pressure; RBC, red blood cell; WBC, white blood cell; HGB, hemoglobin; PLT, platelet; RDW, red blood cell volume distribution width; RDW, red blood cell volume distribution width; HCT, hematocrit; APTT, activated partial thromboplastin time; PT, prothrombin time; INR, international normalized ratio; BE, buffer excess; CRE, creatinine; BUN, blood urea nitrogen; MV, mechanical ventilation; GCS, Glasgow coma score; SOFA, sepsis related organ failure assessment; APSIII acute physiology score III; Blood Transfusion: defined as RBC, Plasma, PLT product administered; Hyperosmolar therapy: defined as HTS or mannitol; Neurosurgical intervention: defined as craniectomy or ventriculostomy*.

### Variable Importance

**Figure 2:**
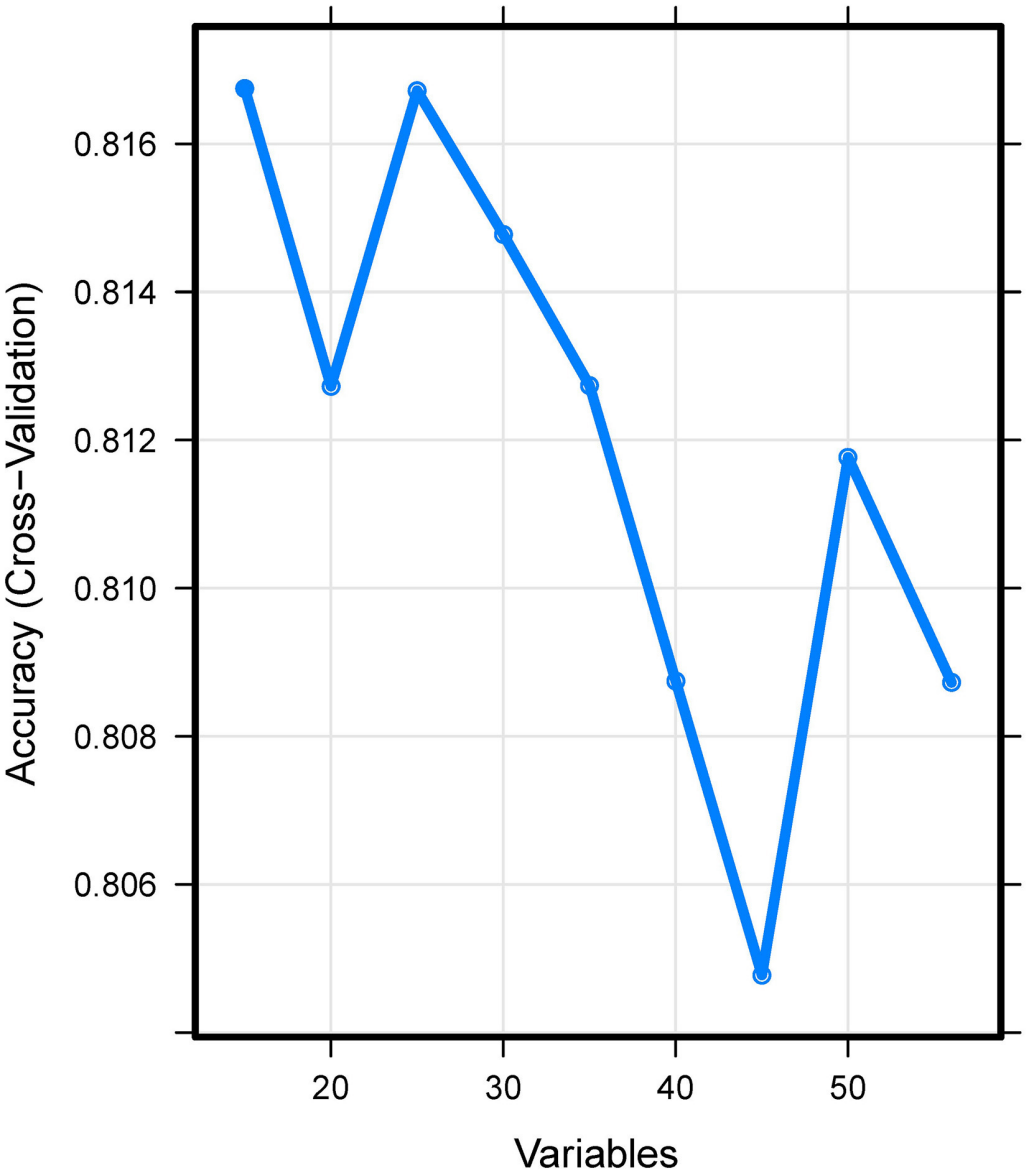
Association between the number of variables allowed to be considered at each split and the prediction accuracy in the REF algorithm. REF, recursive feature elimination.

A total of 15 important predictors (Figure 2) was selected by the RFE algorithm, including INR, PT, SOFA, APTT, PLT, HCT, RBC, HGB, BUN, RDW, CRE, congestive heart failure, myocardial infarction, sodium, and blood transfusion. Then, these 15 variables were used in all the subsequent analysis for all models in both training and testing sets.

### Prediction Performance in eICU-CRD

**Figure 3:**
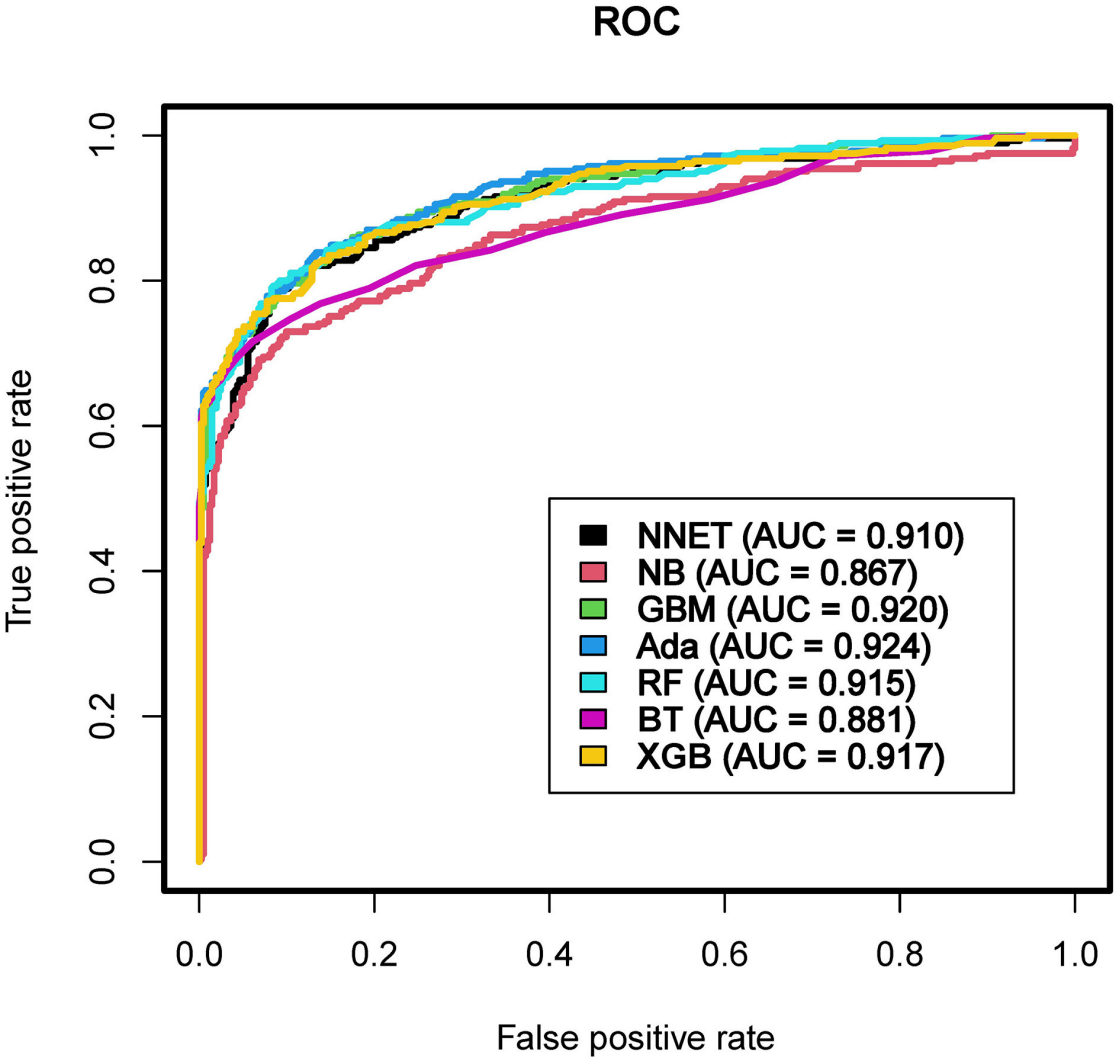
Area under the curve of receiver operating characteristic curve by machine learning models in the validation cohort. ROC, receiver operate characteristics; AUC, area under the curve; NNET, artificial neural network; NB, naïve bayes; GBM, gradient boosting machine; Ada, adapting boosting; RF, random forest; BT, bagged trees; XGB, eXtreme Gradient Boosting.

**Figure 4:**
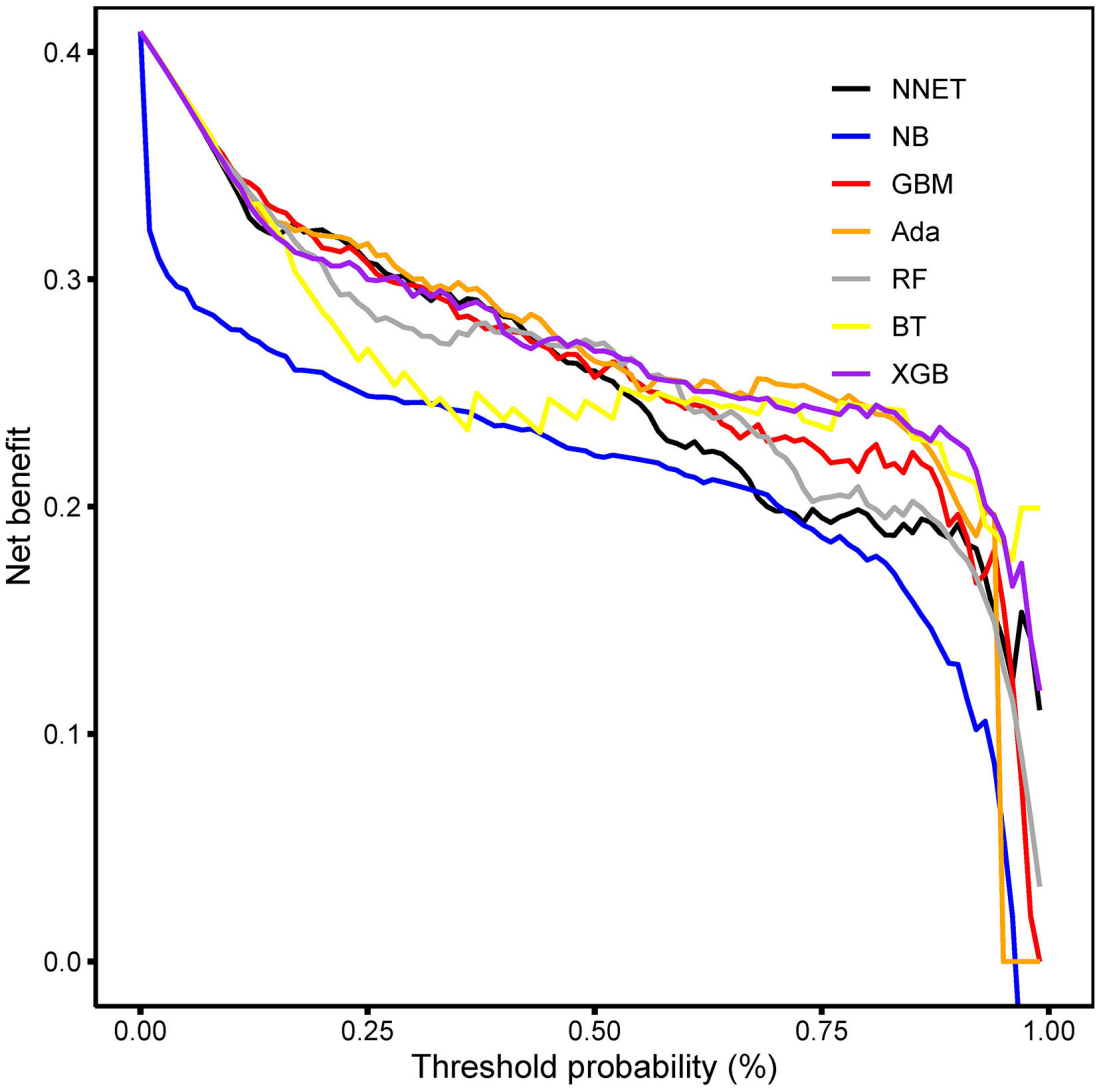
Decision curve analysis. The net benefits of the Ada and XGB are relatively larger over a range of threshold probability values compared with those of other ML models. Ada, adapting boosting; XGB, eXtreme Gradient Boosting; ML, machine learning. NNET, arti?cial neural network; NB, naïve bayes; GBM, gradient boosting machine; RF, random forest; BT, bagged trees.

The discriminatory abilities of all models for the prediction of coagulopathy are in Figure 3 and Table 2. Within the training set, the NNET, NB, GBM, Ada, RF, BT and XGB models were established, and the testing set obtained AUCs of 0.910, 0.867, 0.920, 0.924, 0.915, 0.881, and 0.917, respectively. Comparatively, Ada had the highest predictive performance among these seven models (AUC 0.924, 95% Confidence Interval (CI) 0.902 to 0.943), while NB had the poorest generalization ability (AUC 0.867, 95% CI 0.839 to 0.891). The decision curve compared the net benefit of the best model and alternative approaches for clinical decision making. As shown in Figure 4, the net benefits of the Ada model and XGB model surpassed those of other ML models, including NB for all threshold values, showing that these two models were more superior in predicting the TBI-IC in this cohort.

**Table 2:** Prediction performance of the machine learning models in the testing set.

**Model**	**Accuracy**	**Sensitivity**	**Specificity**	**PPV**	**NPV**	**AUC**	**95% CI**
NNET	0.851	0.733	0.932	0.882	0.835	0.910	(0.886, 0.930)
NB	0.814	0.586	0.971	0.933	0.772	0.867	(0.839, 0.891)
GBM	0.848	0.800	0.881	0.823	0.864	0.920	(0.897, 0.939)
Ada	0.855	0.730	0.942	0.897	0.834	0.924	(0.902, 0.943)
RF	0.862	0.797	0.908	0.857	0.866	0.915	(0.892, 0.935)
BT	0.835	0.747	0.896	0.832	0.837	0.881	(0.854, 0.904)
XGB	0.859	0.744	0.939	0.895	0.841	0.917	(0.894, 0.936)

*PPV, positive predictive values; NPV, negative predictive values, AUC, area under the curve; CI, confidence interval; NNET, artificial neural network; NB, naïve bayes; GBM, gradient boosting machine; Ada, adapting boosting; RF, random forest; BT, bagged trees; XGB, eXtreme Gradient Boosting*.

**Figure 5:**
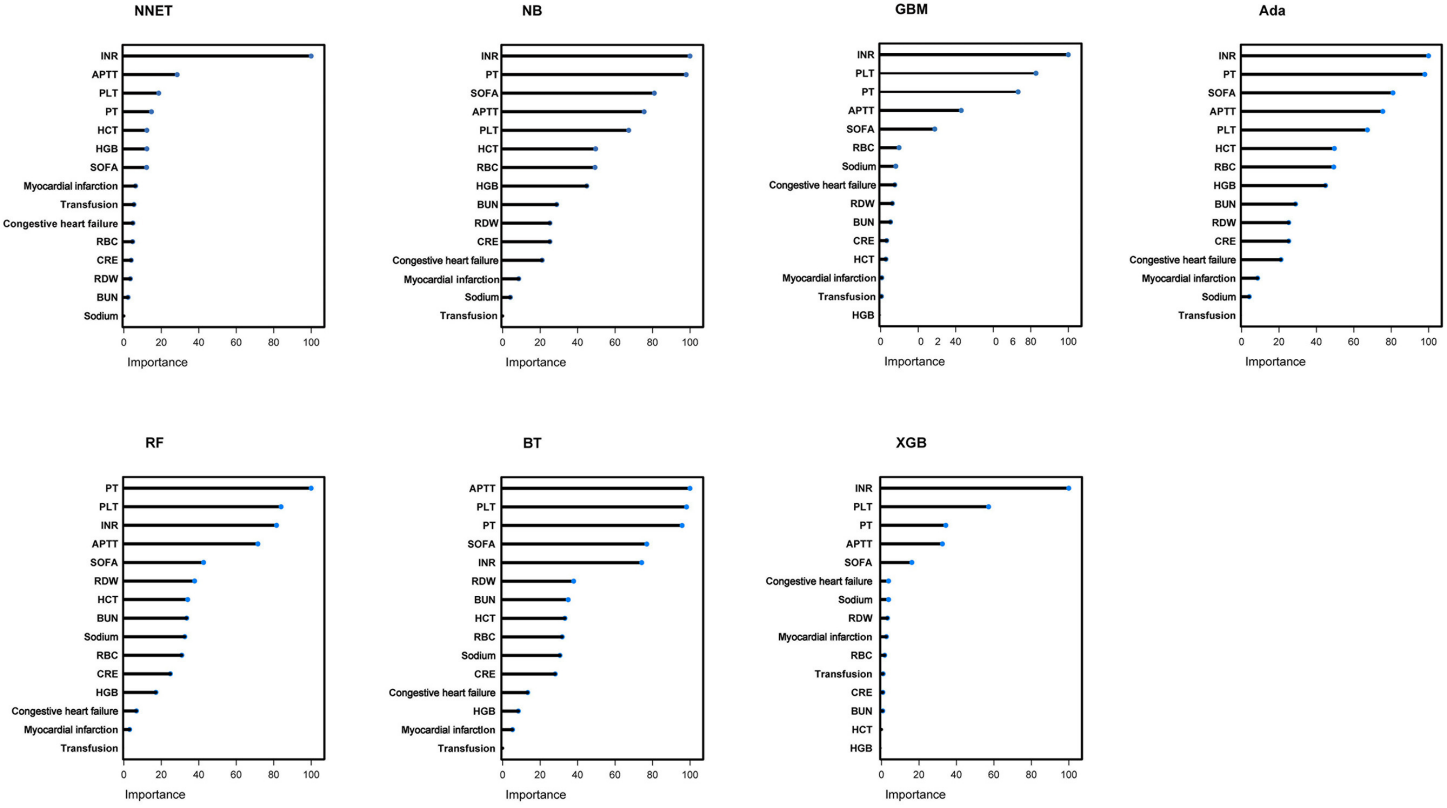
Variable importance in seven different ML models. ML, machine learning; NNET, artificial neural network; NB, naïve bayes; GBM, gradient boosting machine; Ada, adapting boosting; RF, random forest; BT, bagged trees; XGB, eXtreme Gradient Boosting; INR, international normalized ratio; PT, prothrombin time; SOFA, sepsis related organ failure assessment; APTT, activated partial thromboplastin time; PLT, platelet; HCT, hematocrit; RBC, red blood cell; HGB, hemoglobin; BUN, blood urea nitrogen; RDW, red blood cell volume distribution width; CRE, creatinine.

In the Figure 5, the fifth predictor variables in the ML models are shown. Each variable included in the study had varying importance over the TBI-IC relying on the ML approach. Overall, the coagulation profile (PLT, INR, PT) was the variable with relatively higher importance across all ML algorithms, followed by APTT, SOFA, and so forth.

### Model Interpretation

**Figure 6:**
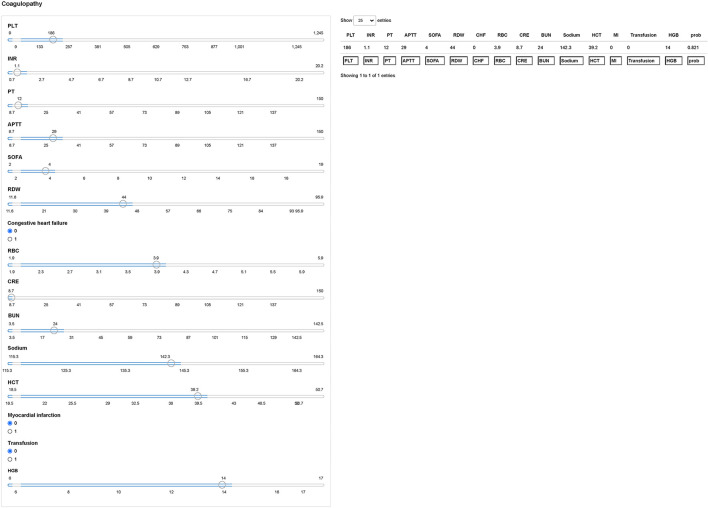
Examples of website usage. Entering the input value determined the coagulopathy and displayed how each value contributed to the prediction. PLT, platelet; INR, international normalized ratio; PT, prothrombin time; APTT, activated partial thromboplastin time; SOFA, sepsis related organ failure assessment; RDW, red blood cell volume distribution width; RBC, red blood cell; CRE, creatinine; BUN, blood urea nitrogen; HCT, hematocrit; HGB, hemoglobin.

We next used the Shiny to illustrate the impacts of key features on the coagulopathy prediction model in individual patients. As shown in Figure 6, the information of one patient was input into the model: PLT (186 × 10^9^/L), INR (1.1), PT (12s), APTT (29s), SOFA (4), RDW (44%), no congestive heart failure, RBC (3.9 × 10^9^/L), CRE (8.7 mg/dL), BUN (24 mg/dL), sodium (142.3 mmol/L), HCT (39.2%), no myocardial infarction, no blood transfusion, HGB (14 g/dl). The model analyzed that the risk of coagulopathy in this patient was 82.10%, indicating that the probability of coagulopathy for the patients was high, and precaution measures were recommended.

## Discussion

Altered hemostasis and hemorrhagic progression are substantial challenges in the clinical management of TBI. Patients with TBI-IC were at a high risk of death over those with normal coagulation. Notably, studies elucidating the rapid prediction of TBI-IC, are warranted. In this sense, our study developed and validated ML models, providing an accurate predictive tool for coagulopathy in TBI patients. Specifically, seven ML models (NNET, NB, GBM, Ada, RF, BT and XGB) were used to predict TBI-IC using variables frequently used in clinical practice. Concerning the predictive performance, the Ada outperformed the remaining models. Moreover, results from the DCA indicated that the Ada and XGB models had higher net benefits over a range of threshold probability values than other models. It is remarkable that this study combined preoperative characteristic, comorbidities, and laboratory findings other than coagulopathy profile to establish a prediction model.

To help surgeons use the model, a calculator was developed, which provided a user-friendly interface. After entering the variables, the incidence of TBI-IC will be shown. The explanation of the ML model at the individual level was consistent with the aforementioned explanations at the feature level, and gratifyingly, the black-box concern was further mitigated to a certain extent. Notably, these results facilitated correct clinical decisions, and more importantly, timely treatment strategy.

A previous study conducted by Cosgriff et al. (20) developed a simple score to predict traumatic brain injury-induced coagulopathy (TIC) using four binary predictors [systolic blood pressure <70 mm Hg, temperature <34°C, pH <7.1, and Injury Severity Score (ISS) >25]. However, due to the fact that the ISS cannot be obtained at the time of decision making, the application of such a score was limited. To predict TIC more accurately, two scores have been developed by prehospital information (21, 22). Mitraet al.'s score used 5 predictors (entrapment; systolic blood pressure < 100 mm Hg; temperature < 34°C; suspected abdominal or pelvic injury; and chest decompression), whereas Peltan et al.'s score employed 6 predictors (age, injury mechanism, prehospital shock index>= 1, GCS, and need for prehospital tracheal intubation and/or Cardiopulmonary Resuscitation (CPR)) (21, 22). Nevertheless, in new patients, both scores achieved only moderate performance, with sensitivity <30%. Additionally, the Trauma Induced Coagulopathy Clinical Score (TICCS) employed three components, including general severity, blood pressure, and extent of significant injuries to predict TIC (23). A major limitation of above scores was that much of the prognostic potential of available information was lost through limiting the number of predictors and dichotomizing continuous variables. Consequently, a novel predictive model for early-identification of TIC was established (Predictors: heart rate, systolic blood pressure, temperature, hemothorax, Focused Assessment with Sonography for Trauma (FAST) result, unstable pelvic fracture, long bone fracture, GCS, lactate, base deficit, pH, mechanism of injury, energy) (24). However, one point worth noting was that previous study focused on the entire trauma patient, not TBI patients in particular, which added confusion to some extent.

By interpreting the full model, it was found that many clinical variables can contribute to predict the risk of TBI-IC. In this study, coagulopathy profile (INR, PT, APTT) was found to be the most important variable in predicting TBI-IC, followed by SOFA, blood routine test (PLT, RBC, HCT, HGB, RDW), renal function (BUN and CRE), comorbidities (congestive heart failure, myocardial infarction) and so forth. Among the fifteen included variables, the SOFA was an important predictor. SOFA is an indicator to describe multiple organ dysfunction, including respiratory system, nervous system, cardiovascular system, liver, coagulation and kidney (25). Potential mechanisms may include the fact that SOFA scores are more likely to indicate liver failure or cardiovascular failure. Those organ failures have a high tendency to bleed, and subsequently leading to coagulopathy (26).

In this study, PLT, RBC, HCT, HGB and RDW were important predictors of TBI-IC. In a prospective observational study conducted by Davis PK et al. (27), PLT dysfunction was an early marker for TBI-IC. Potential mechanism included the blood dilution arised from the use of coagulation factor products (28). Nevertheless, we cannot exclude the likelihood that the blood coagulation system was activated by the continuous bleeding itself (29).

RDW, a parameter of red blood cell volume, measures the variability in size of circulating erythrocytes. Although primarily used to diagnose different types of anemias, the RDW was also associated with various thrombotic disease processes including venous thromboembolism (VTE) (30, 31).

Although the underlying mechanism is unclear, it is speculated that inflammatory factors destroy the vascular endothelial integrity, subsequently changing the glycoprotein and ion channel structure of the erythrocyte membrane (32, 33). Consequently, the deformability of the RBC is reduced, in turn, further enables endothelial damage to increase, causing the release of tissue factors that activate the coagulation pathway and triggers disseminated intravascular coagulation (DIC) (34).

In this study we found that renal function indicators (BUN and CRE) can help to indicate the risk of TBI-IC. Similarly, a ML model developed by Zhao QY et al. also identified renal function, including urine output and CRE to predict sepsis-induced coagulopathy (SIC) (35). It is worth noting that renal dysfunction has been associated with both thrombotic and hemorrhagic complications (36, 37). Potential mechanism included less adenosine diphosphate (ADP) and serotonin storage in PLT of patients with renal dysfunction (38, 39). Taken together, the force of impact at the time of TBI can cause shearing of large and small vessels, and result in subdural, subarachnoid, or intracerebral hemorrhages, or a combination of different types. TBI-associated factors might then alter the intricate balance between bleeding and thrombosis formation, leading to coagulopathy (9). Indeed, the complex interactions between the PLT dysfunction, changes in endogenous procoagulant, anticoagulant factors, endothelial cell activation, hypoperfusion, and inflammation related to TBI-IC remain to be elucidated (9, 40, 41).

The strengths of this study lied in the fact that it applied modern ML approaches to predict TBI-IC. It is worth noting that early and accurate prediction of TBI-IC can provide more time for clinicians to adjust corresponding treatment strategies. For example, this model is applicable if detailed medical history is not available for intubated severe head-injured ICU patient. Furthermore, given the heterogeneity of TBI-IC phenotypes (bleeding/thrombotic tendencies), timely treatment strategy would still require investigation and further testing to determine the type and therefore appropriate treatment. Furthermore, it was based on a real-world data with multicenter and external validation, which heighted the reliability of the performance of ML models. Besides, all the information in this dataset was coded independently of the practitioner, making it a reliable source.

Our study had limitations, consistent with those inherent to many large administrative database studies. First, only TBI-IC adults in ICUs were included, while TBI-IC children and hospitalized TBI-IC cases were not analyzed. Nevertheless, in light of the immaturity of the coagulation system in children, more research is indeed required. Second, derived from the ICU participants, the results of our study cannot be generalized to other population, and we did not obtain information including laboratory testing and interventions before ICU admission, which may cause confounders to some extent. Although our models can screen out patients who are at a high risk of TBI-IC, it is the surgeons who decide the administration of anticoagulant therapy. Usually, the interventions are time sensitive and need to occur early after admission, starting in the emergency department. Third, some new coagulation markers, for example, thrombin-antithrombin-III complex and plasmin-α2-antiplasmin complex, are useful in coagulopathy diagnosis (42, 43). Nevertheless, these indicators were not recorded in the MIMIC-IV and eICU database. This was also the case for viscoelastic coagulation testing [Thrombelastograghy (TEG), Rotational thromboelastometry (ROTEM), ClotPro]. Although these testings can provide detailed coagulopathy diagnosis rapidly and have multiple advantages over the traditional plasma-based coagulation tests (PT, APTT, INR), unfortunately, the above indicators were not included in these two databases. Fourth, we did not obtain the results of cranial Computer Tomography (CT) scans in this study, consequently, the original Corticosteroid Randomization After Significant Head Injury (CRASH)-CT score was not available. Moreover, as an administrative database, there was possibility for misclassification of TBI, to reduce bias caused by imprecise coding, we adopted the extensively used ICD-9, 10 codes. Fifth, as with all potential retrospective studies, there was a potential for unmeasured confounders, causing selection bias. Another major limitation worth noting was the changing nature of the variables in a critically ill patient from time of injury and right throughout the continuum of care to ICU discharge. The nature of the retrospective database did not allow for correction for when measurements were taken in relation to the time of injury. Lastly, although our study deeply explored the coagulopathy of TBI in the ICU settings, other outcomes, such as long-term incidence, are also needed further investigation.

## Conclusions

In general, the present study suggested that some important features were potentially related to the TBI-IC. The ML model processed large number of variables and subsequently discriminated TBI patients who would and would not develop coagulopathy, facilitating the implement of timely yet efficient treatments. In the future, further validation regarding its clinical application value will become a necessity.

## Data Availability Statement

The datasets presented in this study can be found in online repositories. The names of the repository/repositories and accession number(s) can be found below: These data can be found here: https://mimic-iv.mit.edu/; https://eicu-crd.mit.edu/.

## Ethics Statement

The studies involving human participants were reviewed and approved by the Institutional Review Boards of Beth Israel Deaconess Medical Center (Boston, MA) and the Massachusetts Institute of Technology (Cambridge, MA). Written informed consent for participation was not required for this study in accordance with the national legislation and the institutional requirements.

## Author Contributions

FY, CP, and LP: Conception and design. YL: Administrative support. JW: Collection and assembly of data. FY and CP: Data analysis and interpretation. All authors: Manuscript writing and final approval.

## Conflict of Interest

The authors declare that the research was conducted in the absence of any commercial or financial relationships that could be construed as a potential conflict of interest.

## Publisher's Note

All claims expressed in this article are solely those of the authors and do not necessarily represent those of their affiliated organizations, or those of the publisher, the editors and the reviewers. Any product that may be evaluated in this article, or claim that may be made by its manufacturer, is not guaranteed or endorsed by the publisher.

## Supplementary Material

Supplementary Table 1 Missing number (%) for included variables in the dataset.

Supplementary Table 2 Baseline characteristics of the eICU-CRD cohorts.
