# Supplementary material for: A Machine Learning Approach for the Prediction of Traumatic Brain Injury Induced Coagulopathy
Source: Front Med (Lausanne). 2021 Dec 10;8:792689. doi: 10.3389/fmed.2021.792689 (PMC8703138; doi:10.3389/fmed.2021.792689)
Supplement: Supplementary Table 1 — Missing number (%) for included variables in the dataset. [file Data_Sheet_1.docx]

| Supplementary table 1 Missing number (%) for included variables in the dataset | |
| --- | --- |
| Variables | Missing, N (%) |
| Temperature (°C) | 0.88 |
| MAP (mmHg) | 2.83 |
| Heart rate (min) | 0.41 |
| Respiratory rate (min) | 1.00 |
| RBC (×10^9^/L) | 0.59 |
| WBC (×10^9^/L) | 0.59 |
| Hemoglobin (g/dL) | 0.59 |
| PLT (×10^9^/L) | 0.59 |
| RDW (%) | 2.42 |
| HCT (%) | 0.59 |
| pH | 23.76 |
| Bicarbonate (mmol/L) | 1.59 |
| Lactate (mmol/L) | 33.08 |
| BE (mmol/L) | 29.48 |
| Anion gap (mmol/L) | 8.61 |
| PaO_2_ (mmHg) | 22.88 |
| PaCO_2_ (mmHg) | 22.88 |
| FiO_2_ (%) | 29.54 |
| PaO_2_/FiO_2_ | 30.78 |
| Chloride (mmol/L) | 0.35 |
| Calcium (mmol/L) | 1.36 |
| Sodium (mmol/L) | 0.35 |
| Potassium (mmol/L) | 0.35 |
| Glucose (mmol/L) | 0.35 |
| CRE (mg/dL) | 0.35 |
| BUN (mg/dL) | 0.35 |
| Urine output (mL) | 8.61 |
| BMI (kg/m^2^) | 24.59 |
| APSIII | 2.83 |

MAP, mean artery pressure; RBC, red blood cell; WBC, white blood cell; PLT, platelet; RDW, red blood cell volume distribution width; HCT, hematocrit; BE, buffer excess; CRE, creatinine; BUN, blood urea nitrogen; BMI, body mass index; APSIII, acute physiology score III

| Supplementary table 2 Baseline characteristics of the eICU-CRD cohorts. | | | |
| --- | --- | --- | --- |
| Variables | eICU-CRD | | |
|  | Coagulopathy  (n = 285) | Non-Coagulopathy  (n = 412) | *P* Value |
| Demographics |  |  |  |
| Age (y), median [Q1, Q3] | 56.00 (33.00, 72.00) | 55.00 (35.00, 73.25) | 0.520 |
| Male, n (%) | 198 (69.47) | 260 (63.26) | 0.106 |
| Race, n (%) |  |  | 0.682 |
| Black | 26 (9.12) | 39 (9.47) |  |
| White | 219 (76.84) | 320 (77.67) |  |
| Hispanic | 16 (5.61) | 15 (3.64) |  |
| Asian | 5 (1.75) | 5 (1.21) |  |
| Others | 19 (6.67) | 33 (8.01) |  |
| BMI (kg/m^2^), median [Q1, Q3] | 26.54 (23.42, 30.41) | 25.81 (22.53, 29.90) | 0.126 |
| Family history of stroke, n (%) | 16 (5.61) | 35 (8.50) | 0.198 |
| Coexisting disorders, n (%) |  |  |  |
| Myocardial infarction | 8 (2.81) | 17 (4.13) | 0.475 |
| Congestive heart failure | 13 (4.56) | 16 (3.88) | 0.804 |
| Peripheral vascular disease | 4 (1.40) | 7 (1.70) | 1.000 |
| Cerebrovascular disease | 19 (6.67) | 39 (9.47) | 0.240 |
| Dementia | 7 (2.46) | 13 (3.16) | 0.754 |
| Chronic pulmonary disease | 15 (5.26) | 20 (4.85) | 0.947 |
| Rheumatic disease | 2 (0.70) | 4 (0.97) | 1.000 |
| Peptic ulcer disease | 4 (1.40) | 2 (0.49) | 0.233 |
| Diabetes | 51 (17.89) | 57 (13.83) | 0.177 |
| Paraplegia | 0 (0.00) | 0 (0.00) |  |
| Renal disease | 19 (6.67) | 11 (2.67) | 0.018 |
| Malignant cancer | 0 (0.00) | 3 (0.73) | 0.274 |
| Severe liver disease | 8 (2.81) | 1 (0.24) | 0.004 |
| Metastatic solid tumor | 2 (0.70) | 1 (0.24) | 0.571 |
| AIDS | 1 (0.35) | 0 (0.00) | 0.409 |
| CCI, median [Q1, Q3] | 2.00 (0.00, 4.00) | 2.00 (0.00, 4.00) | 0.473 |
| Vital signs (1st 24h) |  |  |  |
| Temperature (°C), median [Q1, Q3] | 37.10 (36.70, 37.60) | 37.20 (36.88, 37.60) | 0.071 |
| MAP (mmHg), median [Q1, Q3] | 80.00 (72.00, 87.00) | 84.00 (77.00, 91.00) | < 0.001 |
| Heart rate (min), median [Q1, Q3] | 93.00 (80.00, 107.00) | 84.00 (75.00, 97.00) | < 0.001 |
| Respiratory rate (min), median [Q1, Q3] | 18.00 (16.00, 21.00) | 18.00 (16.00, 20.00) | 0.174 |
| Laboratory findings (1st 24h) |  |  |  |
| RBC (10^9^/L), median [Q1, Q3] | 3.70 (3.20, 4.12) | 4.10 (3.60, 4.40) | < 0.001 |
| WBC (×10^9^/L), median [Q1, Q3] | 12.30 (9.20, 15.70) | 12.80 (10.00, 15.75) | 0.122 |
| HGB (g/dL), median [Q1, Q3] | 11.00 (10.00, 13.00) | 13.00 (11.00, 14.00) | < 0.001 |
| PLT (×10^9^/L), median [Q1, Q3] | 144.50 (117.00, 196.50) | 220.00 (176.50, 265.00) | < 0.001 |
| RDW (%), median [Q1, Q3] | 14.30 (13.60, 15.40) | 13.70 (13.10, 14.60) | < 0.001 |
| HCT (%), median [Q1, Q3] | 32.20 (28.48, 37.00) | 37.10 (32.80, 40.60) | < 0.001 |
| APTT (s), median [Q1, Q3] | 31.40 (28.00, 37.10) | 26.20 (24.00, 29.10) | < 0.001 |
| PT (s), median [Q1, Q3] | 16.80 (14.80, 20.40) | 13.45 (11.90, 14.40) | < 0.001 |
| INR, median [Q1, Q3] | 1.40 (1.20, 1.80) | 1.10 (1.00, 1.20) | < 0.001 |
| pH, median [Q1, Q3] | 7.37 (7.33, 7.42) | 7.40 (7.36, 7.43) | < 0.001 |
| Bicarbonate (mmol/L), median [Q1, Q3] | 22.00 (19.60, 24.00) | 23.30 (21.00, 25.50) | < 0.001 |
| Lactate (mmol/L), median [Q1, Q3] | 2.80 (1.70, 3.70) | 2.00 (1.50, 2.90) | < 0.001 |
| BE (mEq/L), median [Q1, Q3] | -2.00 (-4.60, 0.86) | -0.71 (-3.05, 1.00) | 0.013 |
| Anion gap, (mmol/L) median [Q1, Q3] | 11.50 (9.00, 14.30) | 11.30 (8.78, 13.75) | 0.329 |
| PaO_2_ (mmHg), median [Q1, Q3] | 142.15 (105.19, 197.50) | 157.50 (115.46, 217.38) | 0.029 |
| PaCO_2_ (mmHg), median [Q1, Q3] | 37.30 (34.01, 41.24) | 37.06 (34.00, 41.26) | 0.955 |
| FiO_2_ (%), median [Q1, Q3] | 52.50 (40.00, 70.00) | 50.00 (40.00, 60.00) | 0.011 |
| PaO_2_/FiO_2_, median [Q1, Q3] | 2.95 (2.13, 3.88) | 3.45 (2.54, 4.55) | < 0.001 |
| Chloride (mmol/L), median [Q1, Q3] | 107.50 (103.50, 112.00) | 105.30 (102.00, 108.62) | < 0.001 |
| Calcium (mmol/L), median [Q1, Q3] | 7.15 (6.00, 8.20) | 8.00 (6.90, 8.60) | < 0.001 |
| Sodium, (mmol/L), median [Q1, Q3] | 140.30 (137.80, 142.80) | 139.15 (137.00, 141.30) | < 0.001 |
| Potassium (mmol/L), median [Q1, Q3] | 3.90 (3.60, 4.20) | 3.80 (3.60, 4.10) | 0.036 |
| Glucose (mmol/L), median [Q1, Q3] | 145.65 (123.38, 174.00) | 136.75 (118.00, 159.68) | 0.003 |
| CRE (mg/dL), median [Q1, Q3] | 0.90 (0.80, 1.20) | 0.80 (0.70, 1.00) | < 0.001 |
| BUN (mg/dL), median [Q1, Q3] | 14.50 (11.00, 19.72) | 13.00 (9.50, 18.00) | 0.001 |
| Urine output (mL), median [Q1, Q3] | 2071.00 (1191.00, 3512.50) | 1891.00 (1235.00, 2962.50) | 0.169 |
| Type of injury, n (%) |  |  |  |
| Subarachnoid hemorrhage | 80 (28.07) | 148 (35.92) | 0.037 |
| Cranial extradural hematoma | 18 (6.32) | 24 (5.83) | 0.916 |
| Cerebral contusion | 21 (7.37) | 33 (8.01) | 0.867 |
| Therapy strategy (1st 24h), n (%) |  |  |  |
| MV | 256 (89.82) | 341 (82.77) | 0.012 |
| Blood transfusion | 49 (17.19) | 14 (3.40) | < 0.001 |
| Hyperosmolar therapy | 51 (17.89) | 52 (12.62) | 0.069 |
| Neurosurgical intervention | 29 (10.18) | 46 (11.17) | 0.772 |
| Scoring system |  |  |  |
| GCS | 5.00 (3.00, 8.00) | 6.00 (3.00, 8.00) | 0.012 |
| SOFA | 8.00 (6.00, 10.00) | 6.00 (4.00, 8.00) | < 0.001 |
| APSIII | 65.00 (45.00, 87.00) | 51.00 (35.00, 71.00) | < 0.001 |

eICU-CRD, eICU Collaborative Research Database; BMI, body mass index; AIDS, acquired immunodeficiency syndrome; CCI, Charlson comorbidity index; MAP, mean artery pressure; RBC, red blood cell; WBC, white blood cell; HGB, hemoglobin; PLT, platelet; RDW, red blood cell volume distribution width; HCT, hematocrit; APTT, activated partial thromboplastin time; PT, prothrombin time; INR, international normalized ratio; BE, buffer excess; CRE, creatinine; BUN, blood urea nitrogen; MV, mechanical ventilation; GCS, Glasgow coma score; SOFA, sepsis related organ failure assessment; APSIII acute physiology score III; Blood transfusion: defined as RBC, Plasma, PLT product administered; Hyperosmolar therapy: defined as HTS or mannitol

Neurosurgical intervention: defined as craniectomy or ventriculostomy
